# A transit portal dosimetry method for respiratory gating quality assurance with a dynamic 3D printed tumor phantom

**DOI:** 10.1002/acm2.13560

**Published:** 2022-02-11

**Authors:** Hong Qi Tan, Calvin Wei Yang Koh, Lloyd Kuan Rui Tan, Kah Seng Lew, Clifford Ghee Ann Chua, Khong Wei Ang, James Cheow Lei Lee, Sung Yong Park

**Affiliations:** ^1^ Division of Radiation Oncology National Cancer Centre Singapore Singapore; ^2^ Division of Physics and Applied Physics Nanyang Technological University Singapore Singapore; ^3^ Oncology Academic Clinical Programme Duke‐NUS Medical School Singapore Singapore

**Keywords:** EPID, motion management, phase gating, quality assurance

## Abstract

**Backgrounds:**

Respiratory gating is one of the motion management techniques that is used to deliver radiation dose to a tumor at a specific position under free breathing. However, due to the dynamic feedback process of this approach, regular equipment quality assurance (QA) and patient‐specific QA checks need to be performed. This work proposes a new QA methodology using electronic portal imaging detector (EPID) to determine the target localization accuracy of phase gating.

**Methods:**

QA tools comprising 3D printed spherical tumor phantoms, programmable stages, and an EPID detector are characterized and assembled. Algorithms for predicting portal dose (PD) through moving phantoms are developed and verified using gamma analysis for two spherical tumor phantoms (2 cm and 4 cm), two different 6 MV volumetric modulated arc therapy plans, and two different gating windows (30%–70% and 40%–60%). Comparison between the two gating windows is then performed using the Wilcoxon signed‐rank test. An optimizer routine, which is used to determine the optimal window, based on maximal gamma passing rate (GPR), was applied to an actual breathing curve and breathing plan. This was done to ascertain if our method yielded a similar result with the actual gating window.

**Results:**

High GPRs of more than 97% and 91% were observed when comparing the predicted PD with the measured PD in moving phantom at 2 mm/2% and 1 mm/1% levels, respectively. Analysis of gamma heatmaps shows an excellent agreement with the tumor phantom.

The GPR of 40%–60% PD was significantly lower than that of the 30%–70% PD at the 1 mm/1% level (*p = 0.0064)*. At the 2 mm/2% level, no significant differences were observed. The optimizer routine could accurately predict the center of the gating window to within a 10% range.

**Conclusion:**

We have successfully performed and verified a new method for QA with the use of a moving phantom with EPID for phase gating with real‐time position management.

## 1 INTRODUCTION

Respiratory motion poses a difficulty in delivering accurate doses to the target in the thoracic and abdominal areas.

This problem is exacerbated by the increasing use of more complex treatment modalities, such as intensity modulated radiotherapy (IMRT) and volumetric modulated arc therapy (VMAT). Furthermore, the use of hypofractionated treatment^1^ (stereotactic radiotherapy and stereotactic ablative body radiotherapy) and particle therapy^2^ leave little margin for errors.

In light of this, there are various motion management and mitigation strategies that increase the delivery accuracy to the target while limiting the dose to the organs‐at‐risk. Phase gating is one such strategy where the beam is delivered only when the patient's breathing phase falls within the preselected window, either the full inspiration phase or end‐expiration phase. The end‐expiration phase is usually preferred due to its reproducibility,^3^, ^4^, ^5^ lower tumor motion, and longer dwelling time. Moreover, it has a dosimetric advantage as it irradiates lower lung volumes in general.^5^ Interestingly, there is another study reporting dosimetric advantage in V_20Gy_ in the left lung and V_30Gy_ in both lungs when treating non‐small cell lung cancer in end‐expiration phase using IMRT.^6^

A commonly implemented practice is to use the real‐time position management (RPM) system^7^ (Varian Medical System, USA), where a surrogate marker is placed on the abdomen of the patient^8^ during simulation and treatment. A charged‐coupled device (CCD) camera mounted on the ceiling of the room will track the reflectors on the marker block, which is illuminated by an infrared source, to capture the breathing motion of the patient.

Conventionally, either a prospective or retrospective four‐dimensional computed tomography (4DCT) will be acquired with RPM. The 4DCT obtained is then used to reconstruct the three‐dimensional CT (3DCT) of each respiratory phase^9^ through phase sorting. There are usually 10 phase bins, and it ranges from 0% to 90%, where 50% and 0% (or 100%) represent exhalation (minimum phase) and inhalation (maximum phase), respectively. The desired phases were selected, and planning was done with the average 3DCT of the selected phases.

Phase gating using RPM is noninvasive and is less demanding on the patient as compared to the breath‐hold technique. However, successful phase gating does have its caveats and pitfalls; the patients must be coached to breathe in a “regular and predictable”^10^, ^11^, ^12^ manner so that the algorithm and the predictive filter can predict the phase accurately during actual treatment. An erratic breathing curve will often lead to excessive beam‐holds, which increases the duration of treatment, and in some cases lead to treatment delivery errors. Additionally, pretreatment imaging is crucial as it ensures the moving tumor target lies within the internal target volume (ITV) contoured from the preselected phases of respiratory motion.

Lastly, AAPM TG‐76^13^ emphasized the importance of quality assurance (QA) of equipment when testing for in vivo dosimetry and target localization. Given that phase gated treatment is a dynamic feedback process, it is crucial that the beam is delivered promptly in the right gating window with minimal delay. Only a device that has a periodic driving force is suitable for this type of QA. This periodic driving force is important as it allows for the mimicking of a human's breathing motion, a characteristic which enables the detectors to measure the dose delivered.

At the point of writing this manuscript, there were two commercial devices that were appropriate for this purpose namely QUASAR by MODUS QA^14^ and the Dynamics Thorax Motion phantom by Computerized Imaging Reference Systems, Inc (CIRS).^15^, ^16^ The detectors used in these commercial solutions are ion chambers and Gafchromic films; both of which have their benefits and drawbacks. Ion chambers give immediate feedback but point dose measurement. On the other hand, while film dosimetry can be cumbersome in terms of the scanning protocol and analysis software,^17^ it gives a 2D dose distribution.

Instead of using the readily available commercial solutions, we chose to utilize an electronic portal imaging detector (EPID) for the equipment QA process. EPIDs not only produce a 2D portal dose (PD) distribution, but they also allow for instantaneous feedback. Portal dosimetry has proven to be a sensitive patient‐specific QA tool when it comes to detecting multi‐leaves collimator (MLC) errors,^18^, ^19^ and it also has in vivo dosimetry^20^, ^21^ capabilities. In fact, EPID was used in several respiratory gating studies in an in vivo setting. Berbeco et al.^22^ used the EPID with fiducial marker in the liver to determine the inter‐ and intra‐fractional shifts in a gated 3D conformal therapy. Saito et al.,^23^ Serpa et al.,^24^ and Lin et al.^25^ show the feasibility of using EPID to visualize and quantify the target motions in gated radiotherapy with RPM in Lung cancer.

A QA device comprising a 3D printed tumor phantom placed isocentrically and driven by a motorized programmable stage was designed to take full advantage of the EPID's capabilities. The PD image is acquired, while the phantom is moving, and the resulting image is then compared to the predicted dose image output using our in house algorithm. This comparison is performed using a gamma analysis where the passing rate directly correlates to the phase gating accuracy.

Our proposed solution is a method that is more time‐efficient in performing the QA process than the commercial solution which involves either setting up of the electrometer and ion chamber or performing film analysis. The rest of the manuscript first outlines the design of our QA device and the details of the PD prediction algorithm. After which, it quantifies the performance of our solution using two different sizes of tumor phantom, a sinusoidal curve and realistic patient's breathing curve.

## 2 MATERIALS AND METHODS

### 2.1 QA set‐up and workflow

**FIGURE 1:**
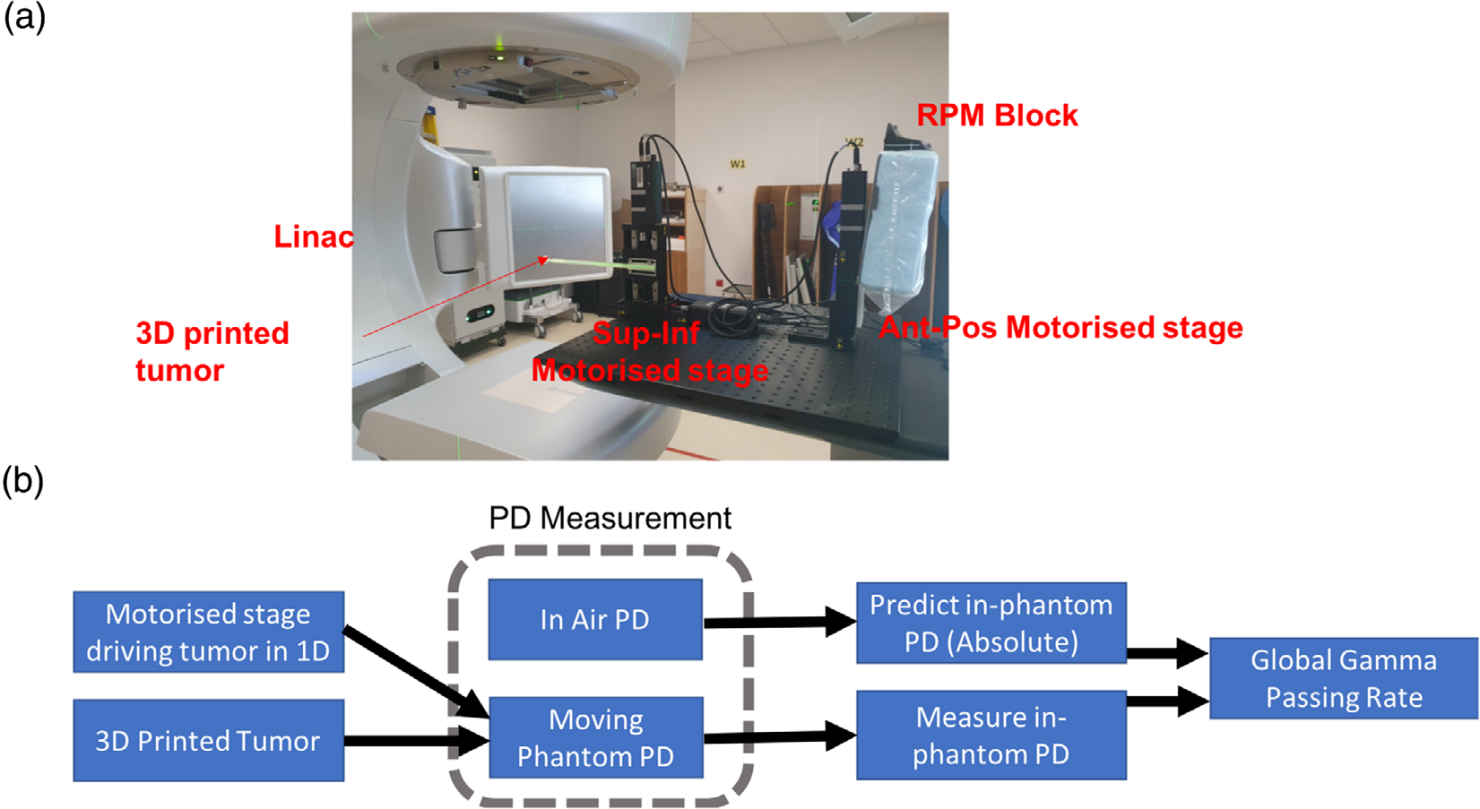
Workflow and photo of the quality assurance (QA) set‐up. (a) Photo of the device set up with electronic portal imaging detector (EPID) imager extended. (b) The proposed workflow for using the QA device. Two PD measurements are required for this workflow – PD in air and PD in moving phantom. The in‐air PD together with the selected gating window, breathing curve, plan, and tumor size is used to generate the predicted PD. Finally, gamma analyses are used to compare the measured PD with predicted PD

A photo of the set‐up of the proposed QA device and the schematics of the QA workflow is shown in Figure 1a,b respectively. The QA setup consists mainly of two components. The first component being the two motorized stages mounted on an optical breadboard. The first stage holds the tumor phantom and is driven in the sup‐inf direction, while the second stage holds the RPM marker block is moved in the ant‐pos direction. The second component being the 3D printed tumor. It is attached to one end of the rod, and the other end is attached to the first motorized stage. The length of the rod is constructed in such a way that it ensures that the couch does not lie with the EPID's field of view when the tumor is placed isocentrically. This rod is printed with a 20% infill to minimize beam attenuation through the rod. The infill density can be interpreted as the “fullness” of the interior of the cuboid with 0% and 100% meaning totally hollow and solid, respectively. We used the amorphous silicon EPID in Truebeam (Varian Medical System, Palo Alto, California) for this study.

The QA workflow starts by identifying the desired gating window from the 4DCT and generating the corresponding treatment plan to deliver the prescribed dose to the ITV. A regular portal dosimetry for patient‐specific QA is then performed to acquire the in‐air PD, $pI_{\mathrm{air}}$. The obtained result serves as a required input for our in‐house algorithm, which is used to predict the PD ($pI_{\mathrm{ph}}$) through a moving tumor phantom. Our algorithm also requires user inputs for the following: the size of the spherical tumor phantom, the selected gating window, and the breathing curve acquired during the simulation phase. The tumor size that best represents the actual tumor should be used. Subsequently the PD measurement is repeated with a moving tumor phantom to yield $pI_{\mathrm{measured}}$. Lastly, the degree of similarity between $pI_{\mathrm{measured}}$ and $pI_{\mathrm{ph}}$ is assessed using the two methods that follow: the first method directly calculates the gamma passing rate (GPR) using both 2 mm/2% and 1 mm/1% criteria, while the second method optimizes the gating window in the algorithm to yield the highest 2 mm/2 mm GPR between $pI_{\mathrm{measured}}$ and $pI_{\mathrm{ph}}$. This entire workflow is illustrated in the schematics in Figure 1b.

### 2.2 3D printing of tumor phantom

The tumor phantom is printed with polylactic acid (PLA) using *method x* 3D printer (MakerBot, Brooklyn, New York). The 3D printer has an enclosure, which enables it to maintain a constant temperature throughout the printing to minimize the chance of warping. In recent years, PLA has been increasingly used in radiotherapy for bolus and QA phantom.^26^, ^27^, ^28^

The infill patterns and percentages were varied to determine the tumor phantom that required the shortest printing time while resembling the Hounsfield units (HUs) of water the most. HUs were determined from the CT scans of a 5 cm x 5 cm x 4 cm printed cuboid. These scans were acquired using a Siemens Somatom Definition scanner with slice thickness of 1 mm. A uniform region of interest (ROI) was drawn within the cuboid. This ROI was then used later when measuring the average and standard deviation of the HU.

Furthermore, measurements were performed to investigate the impact of the print parameters on the dose discrepancy from the predicted dose of 6MV beams calculated by the Eclipse Treatment Planning System (TPS) (Varian Medical System, Palo Alto, California). A 0.6 cc farmer chamber (PTW, Freiburg, Germany) was used for the dose measurement (following the TRS 398 protocol^29^), and it was placed at a depth of 3 cm (water equivalent plastic phantom). The printed cuboid was placed on the surface of the water phantom with the center aligned to the field center.

### 2.3 Motion control with programmable stages

Two motorized closed‐loop linear stages (model X‐LRQxL‐E from Zaber Technologies Inc., Vancouver, British Columbia) were connected in a daisy chain where one stage drives the tumor in the sup‐inf direction, and the other stage moves the RPM block in the ant‐pos direction. The encoder resolution is 500 counts per revolution, and the stage performs 200 motor steps per revolution. These stages have a travel range of 75 mm, repeatability of less than 3 μm and load capacity of 100 kg. It is programmed to either execute a sinusoidal motion or an actual patient's breathing curve data that is output directly from the Varian RPM system.

The input breathing curve data were then sampled at a regular time interval. The position and velocity were calculated and sent as commands to both the stages driving the tumor and the marker block. The user has the option of selecting a different amplitudes or frequencies of the input breathing curve as well as between the two stages. The accuracy of the stage motion for the patient's breathing curve is measured by comparing the encoder output with the actual input breathing curve. This is how the positional accuracy and the time delay are quantified.

### 2.4 Predicting portal dosimetry through phantom

Current commercial TPS is not able to predict PD through a phantom or a patient. As such, an in‐house program based on python 3.6 was developed to generate an average CT of a spherical tumor based on the desired motion's amplitude and gating window. It also proved to be useful in calculating the portal dosimetry results through the “averaged” moving phantom. The portal dosimetry calculation follows the method outlined by Najem et al.,^30^ which calculates the PD through a phantom, $pI_{\mathrm{ph}}$, using the PD obtained in free space, $pI_{\mathrm{air}}$:

(1) $$\begin{array}{ccc} pI_{\mathrm{ph}} & = & pI_{\mathrm{air}}\times T\left(x,y,\mathrm{FS},t\right)\times \mathrm{OAR}\left(x,y,t\right)\\ & & \times \;G\left(x,y,t,\mathrm{FS},g\right) \end{array}$$

x and y refer to the 2‐dimensional spatial position on the EPID, FS represents the equivalent field size,^30^ g is the air gap between the detector and phantom exit, and t is the radiological thickness of the phantom. T, OAR,and G represent the transmission factor, off‐axis ratio factor, and air gap factor, respectively.

The first factor considers the attenuation of photons. The second factor accounts for the different photon spectra through a phantom in the lateral direction. The third factor accounts for the changing air gap between the detector and the exit of the beam through the phantom. The effects of the second and third factors are negligible in this work due to the small phantom dimension.

Spherical tumors are currently used in this work as a proof of concept. The tumors are displaced in the sup‐inf direction based on the breathing curve data from RPM. The final “averaged tumors” are obtained by finding the mean of all the displaced tumors within the gating window (defined using the phase information in the breathing curve data). Since the displaced tumors are generated based on the sampling time of the breathing curve data, the “averaged tumor” takes into account the weighting based on the dwell time; this will not be possible if the “averaged tumor” is obtained from the ten phases in a 4DCT.

Siddon's ray tracing algorithm^31^ is implemented to determine the radiological length of the “averaged moving tumor” as seen by each pixel of the EPID.

The transmission factor T is calculated using the formula and fitting parameters that are defined and obtained by M. Najem et al.:

(2) $$T\left(t,\mathrm{FS}\right)=\frac{A\left(\mathrm{FS}\right)e^{-B\left(\mathrm{FS}\right)t}+C\left(\mathrm{FS}\right)e^{-D\left(\mathrm{FS}\right)t}}{S\left(0,\mathrm{FS}\right)}$$

### 2.5 Comparison of predicted and measured portal dosimetry

The predicted and measured PDs were compared for moving phantom under gated treatment and static phantom under nongated treatment. Two spherical tumors of diameters 2 cm and 4 cm are printed for this measurement study. The tumor sizes are chosen to be less than 5 cm for use in lung stereotactic body radiotherapy (SBRT).^32^ For the moving phantom, PD was measured with each tumor size under three different sinusoidal motion amplitudes (peak to peak) of 1.0, 2.0, and 3.0 cm and two different gating windows of 40%–60% and 30%–70%. The motion amplitudes were chosen to span the range that was encountered in clinic, and the gating windows were chosen to center at the exhalation phase (50%) with the gating width being what we will be using in the clinic. Forty percent to 60% gating window yields a more stationary tumor with smaller ITV but gives a lower duty cycle (higher treatment time). Two different clinical VMAT lung treatment plans (with three fields each) from patients treated in our clinic were used for this study. The dose fractionation and beam energies are the same in both plans, and the differences lie primarily in the field sizes and MLCs configurations. This gives a total of 36 PD measurements for each plan.

The measured and predicted PD was compared using a gamma index analysis^33^, ^34^ at 2 mm/2% and 1 mm/1% levels using 10% dose threshold. These criteria were chosen as it is currently used in our regular patient‐specific quality assurance when using portal dosimetry. The GPR of the static phantom is used for two main purposes. The first is to provide a reference for comparison with the GPR calculated from the predicted and measured PD in the moving phantom's. The second is to measure the alignment errors that arise when using the QA set‐up in practice. Gamma index heatmaps were also plotted for one of the fields for both the static and moving phantom to elucidate the areas of disagreement between $pI_{\mathrm{measured}}$ and $pI_{\mathrm{ph}}$.

GPR is compared between two different gating windows at 2 mm/2% and 1%/1 mm level using Wilcoxon signed‐rank test to test for a significant difference. All reported *p* values are two‐sided and statistically significant if *p <* *0.05*. A repeated plotting of the gamma index heatmaps was done for one of the fields for the two gating windows. The purpose was to highlight the regions where the gamma index of 30%–70% was higher than 40%–60%.

### 2.6 Estimating actual gating window in measurement

An optimizer routine was developed to determine which optimal gating window yields the highest 2 mm/2% GPR between $pI_{\mathrm{measured}}$ and $pI_{\mathrm{ph}}$. This optimizer routine involves a grid search in two‐parameter spaces—the center and width of the gating window. The center is increased by steps of 5% from 0% to 100%, while the half‐width of the window includes 5%, 10%, 15%, and 20%.

The gating window estimation algorithm was tested on a realistic patient's breathing curve acquired during the 4DCT simulation. Both 40%–60% and 30%–70% gating window were chosen, and their PDs were acquired with the moving tumor phantoms of 2 cm and 4 cm. The measurements were also repeated with two VMAT plans. The phase gating accuracy and functionality were previously verified via a dosimetric measurement using a QUASAR phantom. Point dose measurement with a similar farmer chamber yields dose discrepancy of less than 5% from TPS for four different clinical plans (two SBRT plans and two conventional fractionation plan). The algorithm was then applied to the 24 measured PDs to estimate the actual gating window. The difference between the center of the actual and predicted gating windows was used as a figure of merit for comparing their similarity.

It is important for the optimizer to yield a stable gating parameter or well‐defined global maximum in the face of small perturbations, which could arise in measurement. These perturbations could be due to gantry sags, tumor not driven in a perfectly sup‐inf direction or differences in acquired and actual tumor phantom motions. A further investigative work is conducted to determine the theoretical sensitivity of the center and width of the optimal gating window to these perturbations. We assume three different perturbation scenarios consisting of a global 1 mm and ‐1 mm set‐up error, and a normal random error with parameters derived from the positional errors of the linear stages in the sup‐inf direction. These errors are incorporated in the breathing motions and affect the radiological thickness of the phantom, t, and thus the transmission factor, T(x,y,FS,t) in Equation (1). The predicted PD with perturbation, $pI_{\mathrm{perturb}}$, is then calculated with Equation (1), and the optimizer will be used to map the 2 mm/2% GPR across the entire grid search parameters (center and width of the gating window) to find the optimal gating parameters of $pI_{\mathrm{perturb}}$. This entire pipeline is repeated for 4‐cm and 2‐cm tumor sizes, 1 cm and 3 cm peak‐to‐peak motion amplitudes and 30%–70% and 40–60% actual gating windows.

## 3 RESULTS

### 3.1 3D printed tumor phantom

**FIGURE 2:**
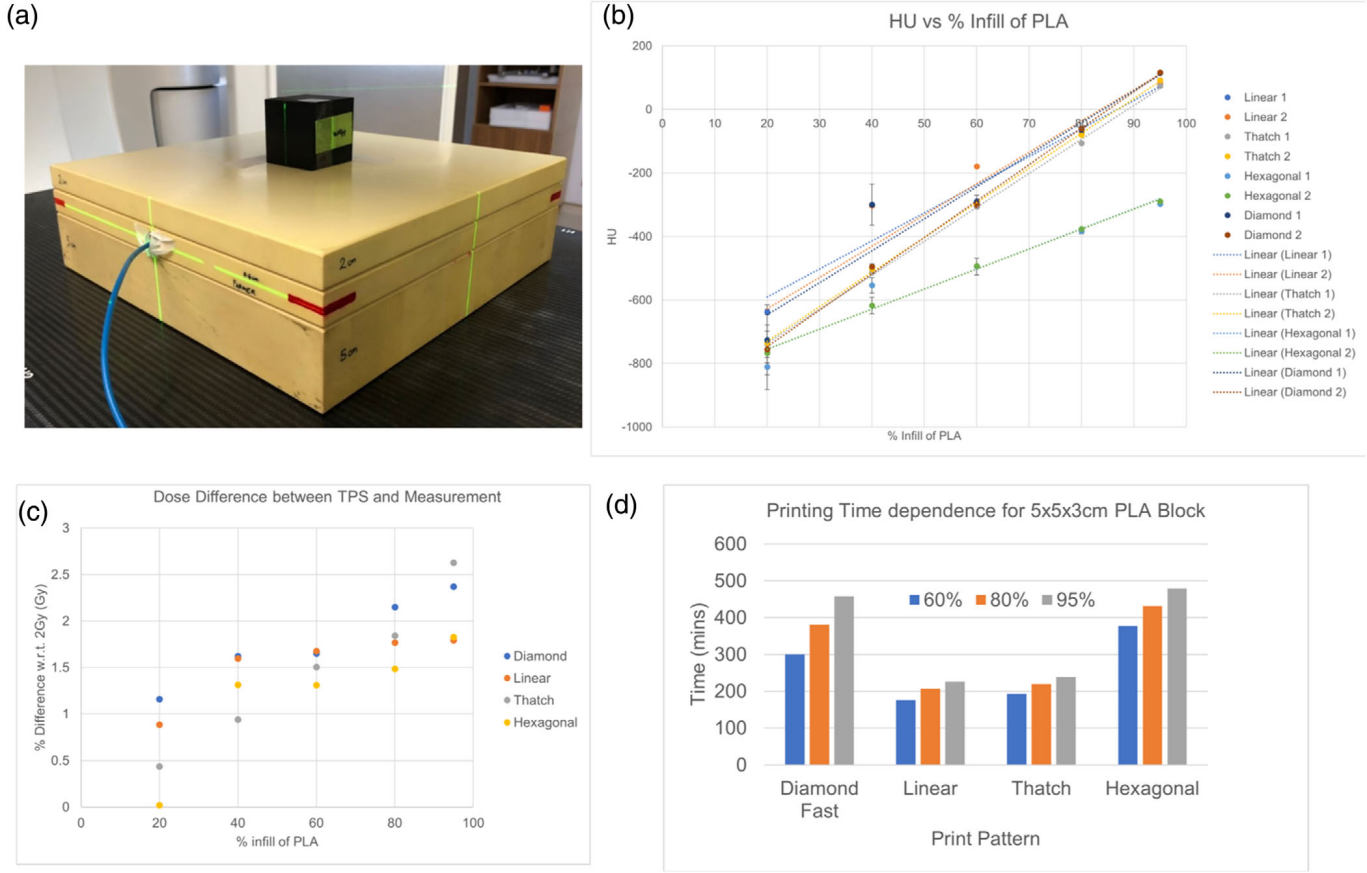
Characterization of 3D printing parameters using Hounsfield units (HU) and dose discrepancy. (a) Photo of the printed cuboid and water phantom to perform dosimetry measurement. (b) Graph of HU against percentage infill of polylactic acid (PLA). The dots are the measurement data, while the lines are the best fit line. (c) Graph of percentage dose difference between measured dose and calculated dose (in Eclipse) against percentage infill of PLA. (d) Bar charts of the printing time against different print patterns

The average HU of the printed cuboid as a function of the percentage PLA infill and the patterns are shown in Figure 2b. The ‐1 and ‐2 suffix in the legend represents the HU obtained while placing the printed cuboid in two different orientations (CT scan direction parallel and perpendicular to the Z direction of the 3D printing). The linear best fit lines are also drawn in the figure to show the gradient, which indicates the rate of increase in HU with percentage infill. The dose measurement set‐up and results are shown in Figure 2a,c, respectively. The percentage dose discrepancy between measurement and Eclipse TPS is observed to increase with percentage infill but is still less than 3%. The TPS dose algorithm used in this work is analytical anisotropic algorithm v13.6 with 2‐mm calculation grid size. The printing time for the different infill patterns and percentages is shown in Figure 2d. The linear pattern phantoms are printed in the shortest time, while hexagonal pattern phantoms take almost twice as long.

### 3.2 Motion control with 3D stages

**FIGURE 3:**
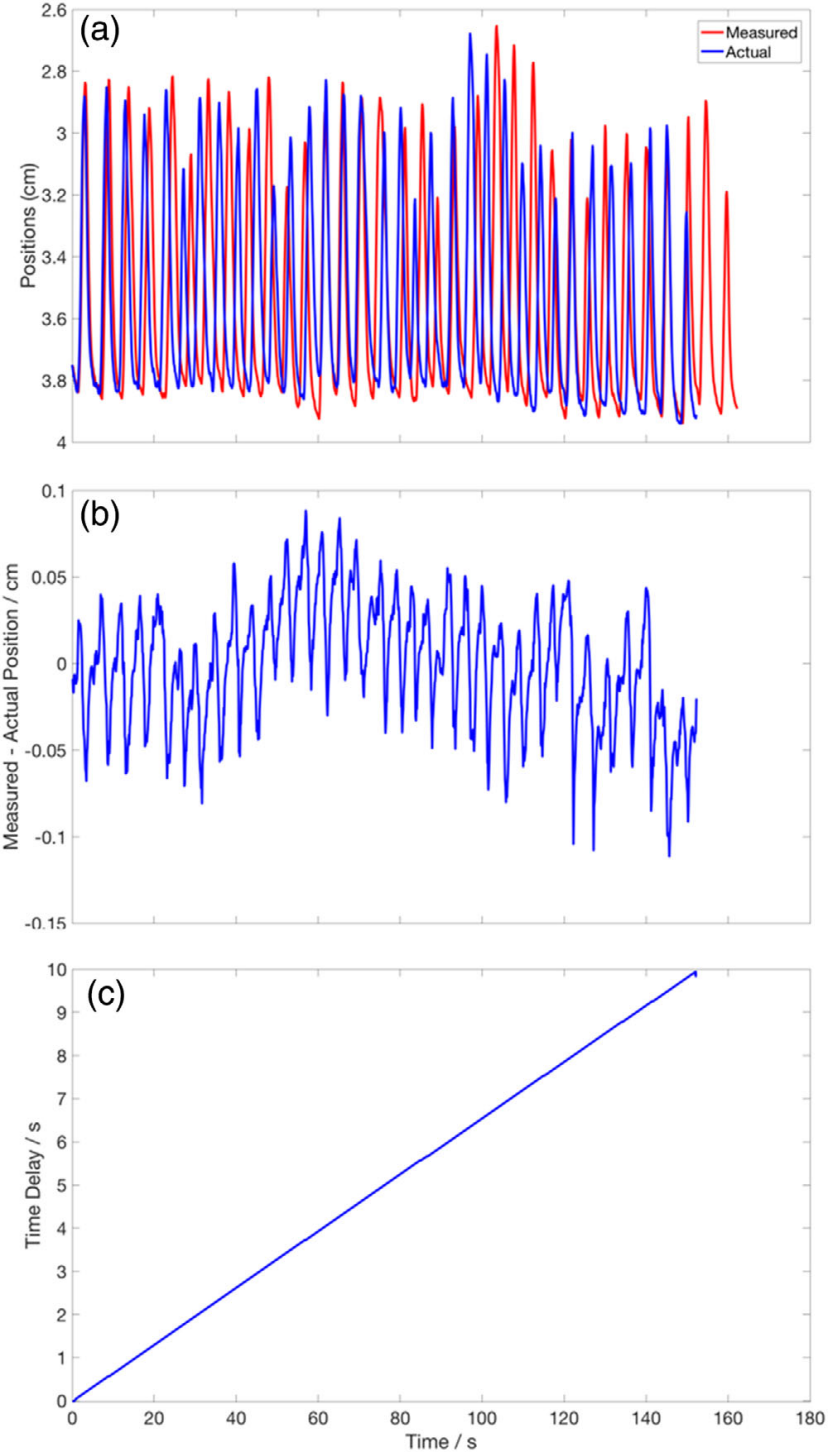
Positional and temporal accuracy of the 2D Motorized stages. (a) A plot of the actual (in blue solid line) and measured (in red solid line) positions of the motorized stage using an actual patient breathing curve. (b) The positional difference between the measured and actual breathing curve. (c) The cumulative time delay in the measured signal over the entire run‐time. The delay is calculated to be 0.0655 s per second of run time (gradient of the line)

Figure 3a shows the comparison of the actual input (in blue line) and the measured breathing curve motion executed by the stage. The positional errors and time delay of the stage as a function of run time are shown in Figure 3b,c, respectively. The mean absolute positional error is (0.0286 ± 0.0206) cm, and the maximal error is up to 0.1 cm. These errors occur at both the maximum and the minimum point of the breathing curve. The time delay has been quantified to be 0.0655 s for each second of run time.

### 3.3 Comparison of predicted and measured portal dosimetry

**FIGURE 4:**
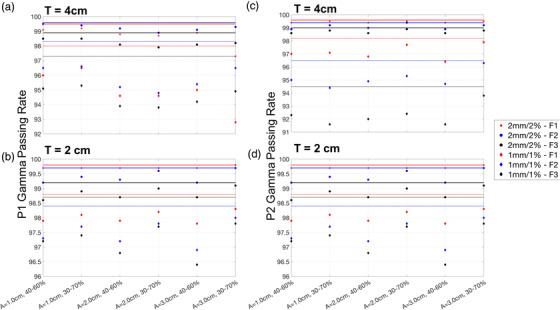
Gamma passing rate of predicted portal dosimetry against measured portal dosimetry in moving phantom moving sinusoidally. These figures show the gamma passing rate for different tumor sizes, motion amplitudes, plans, and gating windows. The motion's amplitudes and gating windows are shown on the x‐axis. The tumor phantom sizes are indicated in the top left‐hand corner of each plot. (a–d) Two different patients’ plans, patient 1 and patient 2, respectively. The circular and diamond markers represent the passing rate at 2 mm/2% and 1 mm/1% levels. The red, blue, and black markers correspond to the three different fields in the plans. The red, blue, and black solid lines are the passing rates for the three fields at the 2 mm/2% level. The red‐, blue‐, and black‐dashed lines are the passing rates for the three fields at the 1 mm/1% level

The overall results on the GPR between the predicted and measured PD are shown in Figure 4. The red, blue, and black markers represent the GPR for fields 1, 2, and 3, respectively. The circular and diamond markers are the results of analysis at 2 mm/2% and 1 mm/1% levels respectively. The red, blue, and black solid lines are the 2 mm/2% GPR of stationary tumor phantom of field 1, 2, and 3. Similarly, the red, blue, and black dotted lines represent the GPR of stationary phantom but at a 1 mm/1% level. These lines act as a reference for comparing the GPR of moving phantom.

Figure 4a,b shows the results of the first plan, while Figure 4c,d shows the result of the second plan. The tumor size used for measurement is indicated in the top left corner of each plot. The GPRs at the 2 mm/2% level are above 97% and 98% for the larger and smaller tumor, respectively. At the 1 mm/1% level, GPRs are above 91% and 96 % for the larger and smaller tumor, respectively. In particular, the small tumor achieves consistently higher GPR than the larger tumor phantom, and the GPR of the stationary tumor (indicated by solid and dotted lines) is the highest. There is no statistically significant difference between the GPRs when comparing across different peak‐to‐peak amplitudes of 1.0, 2.0, and 3.0 cm.

**FIGURE 5:**
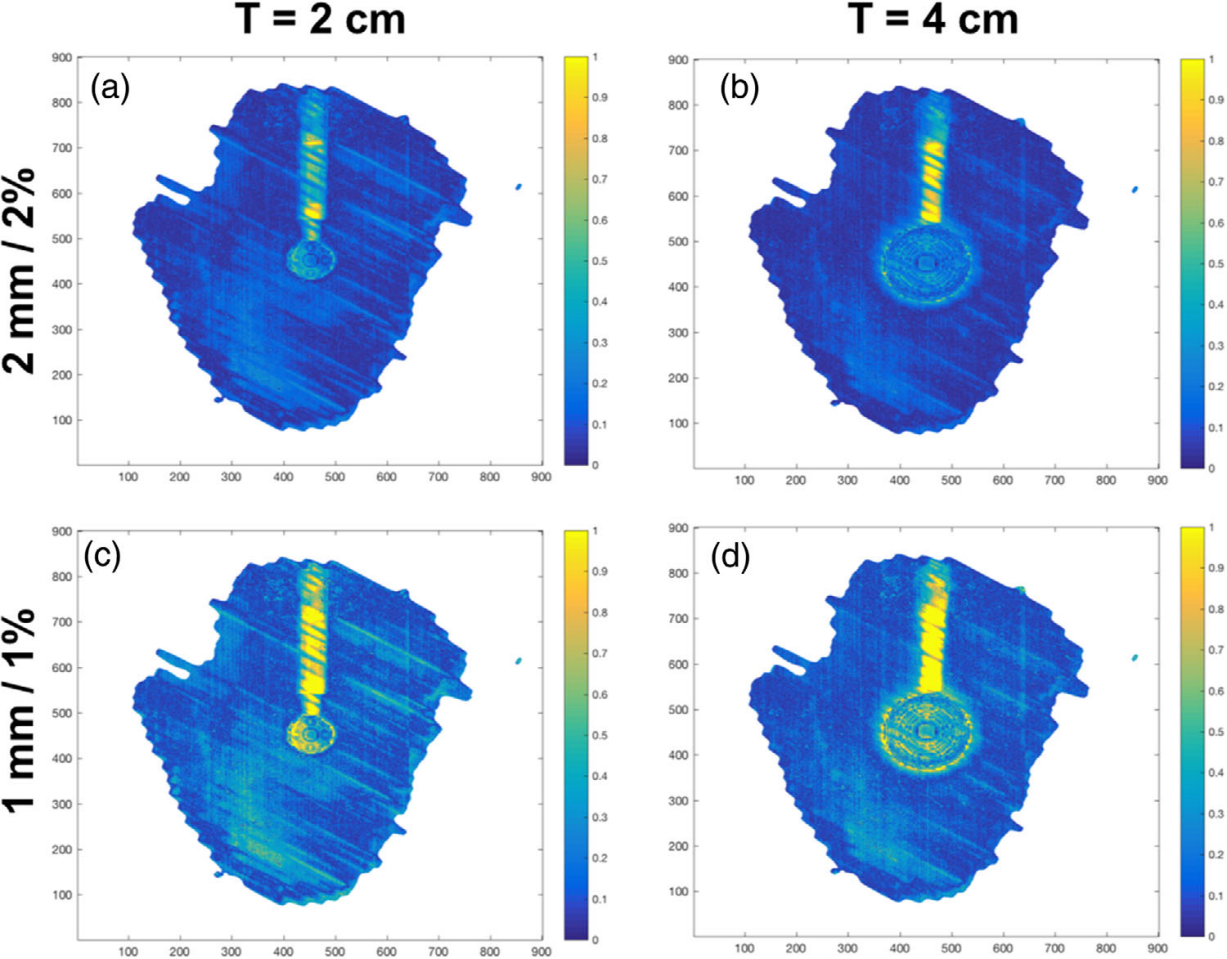
The gamma heatmap of the static phantom at 2 mm/2% and 1 mm/1% level. These figures show the gamma heatmaps of the comparison of $pI_{\mathrm{measured}}$ and $pI_{\mathrm{ph}}$ of the static phantom. (a and c) The heatmaps for the 2 cm phantoms under 2 mm/2% and 1 mm/1% criteria, respectively. (b and d) The heatmaps for the 4 cm phantoms under 2 mm/2% and 1 mm/1% criteria, respectively

Gamma index heatmap of the comparison between $pI_{\mathrm{measured}}$ and $pI_{\mathrm{ph}}$ of the static phantom is shown in Figure 5. Figure 5a,b shows the heatmap for the 2‐cm and 4‐cm tumor phantoms, respectively, with a 2 mm/2% criteria. On the other hand, Figure 5c,d shows the heatmap for the 2‐cm and 4‐cm tumor phantoms, respectively, with a 1 mm/1% criteria. Excellent agreement between $PD_{\mathrm{measured}}$ and $PD_{\mathrm{ph}}$ were observed throughout the entire tumor phantom except for the supporting rod.

**FIGURE 6:**
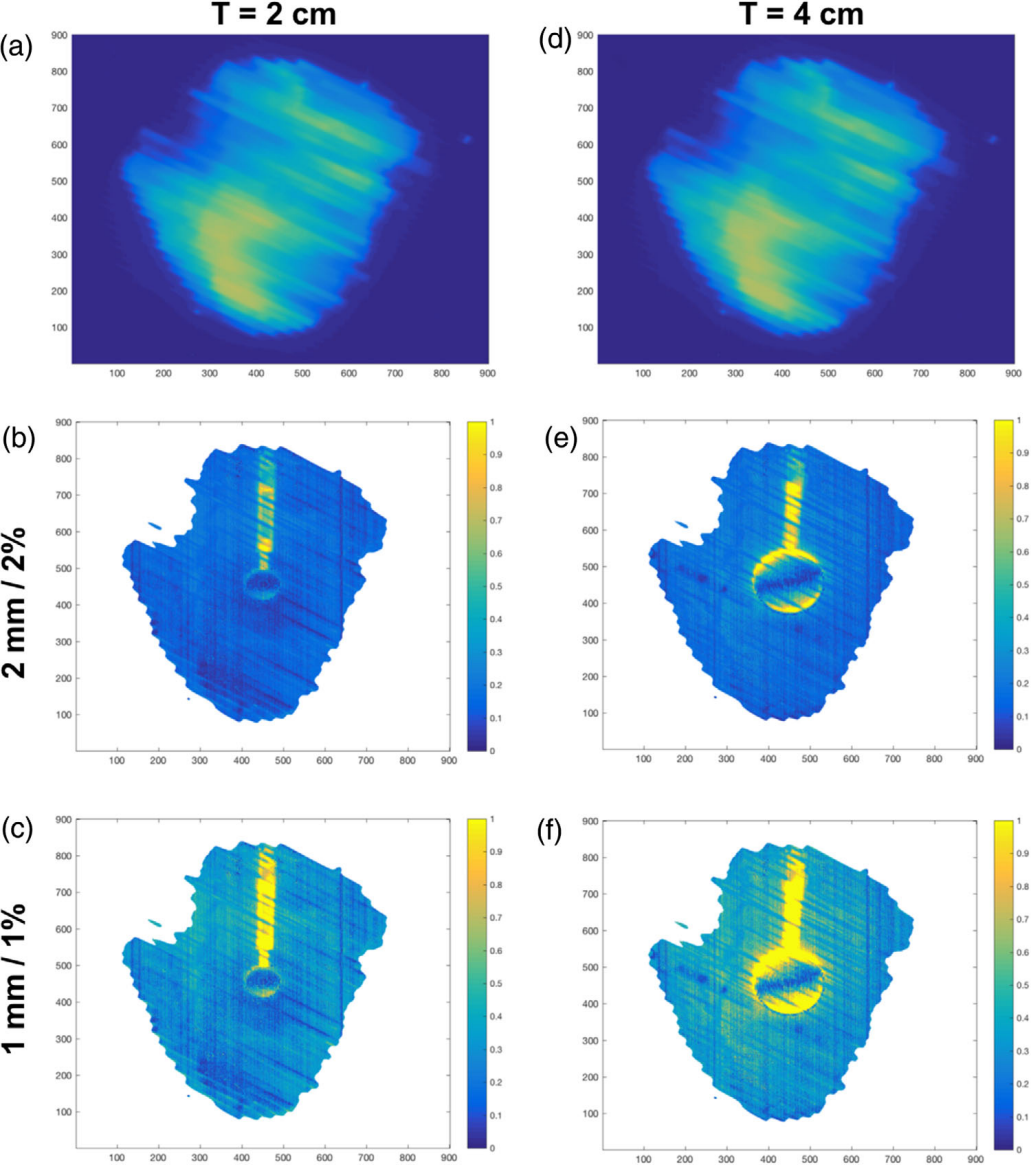
Gamma heatmap of the moving phantom with a 40%–60% gating window under 2 mm/2% and 1 mm/1% criteria. (a and d) The measured portal image acquired with the 2‐cm and 4‐cm phantoms, respectively. (b and c) The heatmaps for the 2‐cm phantoms under 2 mm/2% and 1 mm/1% criteria, respectively. (e and f) The heatmaps for the 4‐cm phantoms under 2 mm/2% and 1 mm/1% criteria, respectively

Figure 6 shows the comparison of the gamma index heatmaps between the small and large tumor for the 40%–60% gating window. Figure 6a–c shows the PD measurement and gamma index heatmaps at the 2 mm/2% and 1 mm/1% level, respectively, for the 2‐cm phantoms. For the 4‐cm phantoms, Figure 6d–f shows the PD measurement and gamma index heatmaps at 2 mm/2% and 1 mm/1% levels. Most of the high gamma index pixels (failing pixels) lie at the sup‐inf edges of the tumor phantom and the supporting rod. The smaller phantom also has fewer regions of failing pixels compared to the larger phantoms.

The Wilcoxon paired rank test for difference in GPR between 40%–60% and 30%–70% gating windows yields *p = 0.9653* for the 2 mm/2%level and *p = 0.0064* for the 1 mm/1% level. Hence, the GPR of the 40%–60% gating window is statistically lower than the 30%–70% window at the 1 mm/1% level.

**FIGURE 7:**
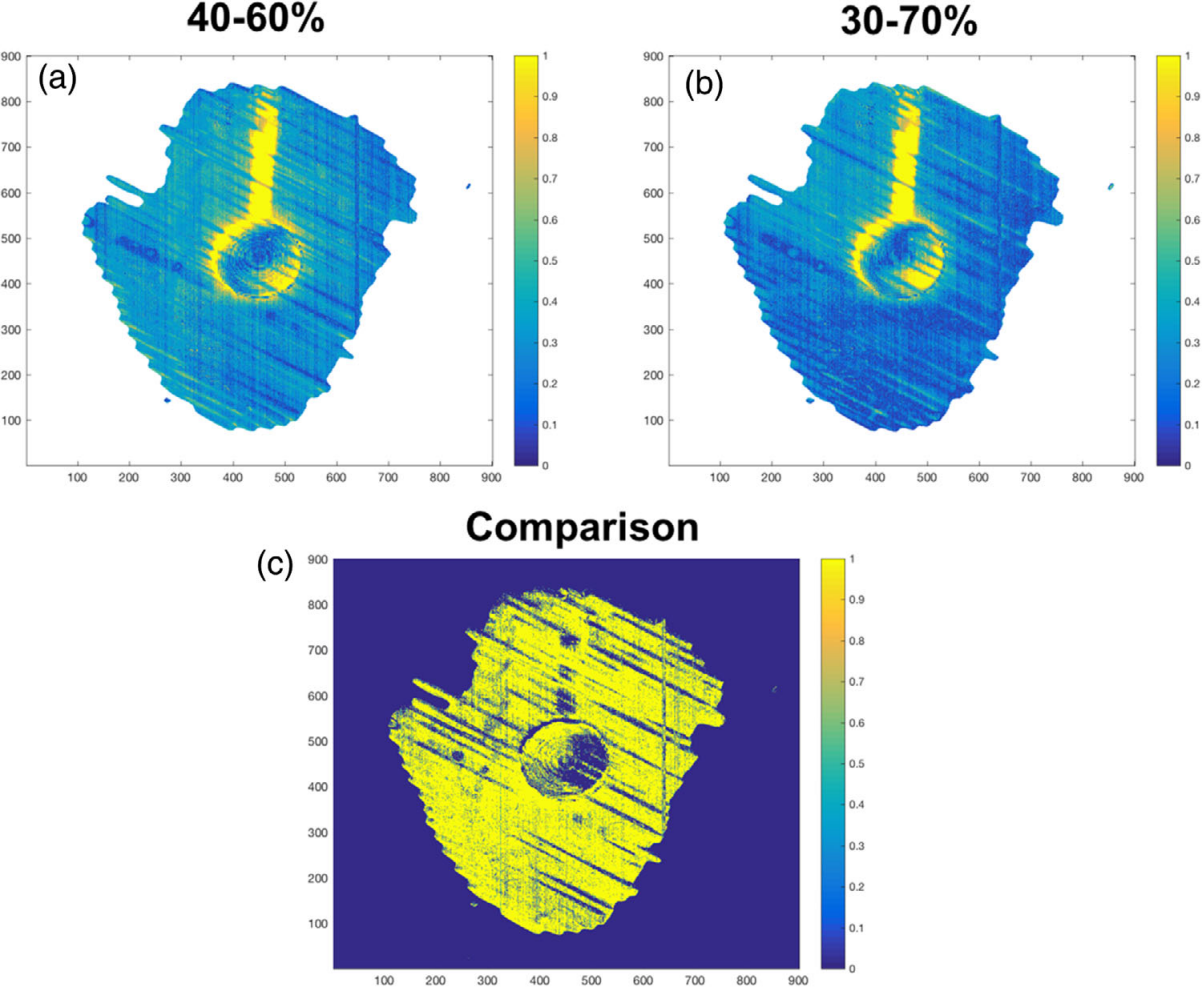
Comparison of gamma heatmap of 40%–60% gating windows and 30%–70% gating windows with 1 mm/1% criteria. (a) Gamma heatmap of a 4 cm moving tumor phantom under 40%–60% gating window and with a 1 mm/1% criteria. (b) Gamma heatmap of a 4 cm moving tumor phantom under 30%–70% gating window and with a 1 mm/1% criteria. (c) The binary map showing the pixels, which have higher gamma values in (a) than in (b)

The 1 mm/1% gamma index heatmap in Figure 7a,b shows that the gamma index is generally higher in all the pixels for the 40%–60% gating window compared to that of the 30%–70% gating window. This is further substantiated in Figure 7c where the highlighted pixels reveal the points that the gamma index is higher for 40%–60% gating window compared to that of the 30%–70% gating window.

### 3.4 Estimating actual gating window in measurement

Results of the estimated gating window from the optimizer algorithm are shown in Table 1 (for 40%–60% actual gating window) and Table 2 (for 30%–70% actual gating window). The difference between the centers of the actual and predicted window is shown in the fifth column of Tables 1 and 2. Comparable differences of 0%–10% in the centers of the gating windows are observed for both the 2‐cm and 4‐cm tumor phantoms. This observation applies to all plans as well as both 30%–70% and 40%–60% gating windows. All the predicted gating windows overlap with the actual preselected gating windows despite the mismatch in widths of predicted window width and the actual window.

**TABLE 1:** Results of the predicted gating windows estimated from the optimizer algorithm

	Field number	Actual gating window	Predicted gating window	Difference in gating
Plan 1, T = 2.0 cm	1	40%–60%	25%–65%	5%
	2	40%–60%	25%–65%	5%
	3	40%–60%	25%–65%	5%
Plan 2, T = 2.0 cm	1	40%–60%	20%–60%	10%
	2	40%–60%	25%–65%	5%
	3	40%–60%	25%–65%	5%
Plan 1, T = 4.0 cm	1	40%–60%	45%–55%	0%
	2	40%–60%	45%–55%	0%
	3	40%–60%	35%–55%	5%
Plan 2, T = 4.0 cm	1	40%–60%	45%–75%	10%
	2	40%–60%	35%–55%	5%
	3	40%–60%	45%–55%	0%

**TABLE 2:** Results of the predicted gating windows estimated from the optimizer algorithm

	Field number	Actual gating window	Predicted gating window	Difference in gating
Plan 1, T = 2.0 cm	1	30%–70%	40%–80%	10%
	2	30%–70%	25%–65%	5%
	3	30%–70%	25%–65%	5%
Plan 2, T = 2.0 cm	1	30%–70%	20%–60%	10%
	2	30%–70%	40%–80%	10%
	3	30%–70%	25%–65%	5%
Plan 1, T = 4.0 cm	1	30%–70%	45%–55%	0%
	2	30%–70%	45%–55%	0%
	3	30%–70%	50%–70%	10%
Plan 2, T = 4.0 cm	1	30%–70%	25%–55%	10%
	2	30%–70%	35%–55%	5%
	3	30%–70%	45%–55%	0%

**FIGURE 8:**
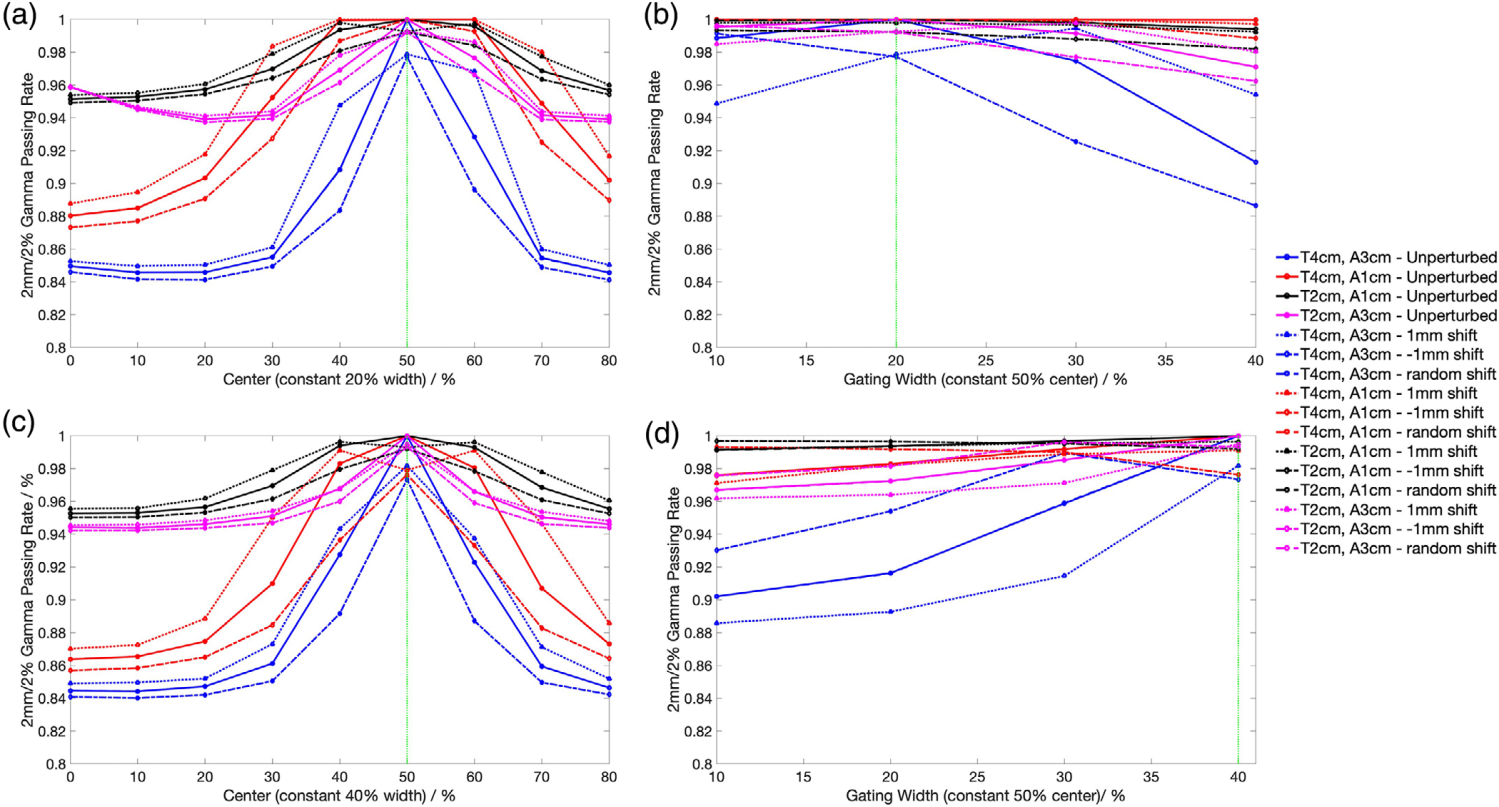
Mapping the gamma passing rate (GPR) as a function of gating width and center during optimization with the introduction of perturbation scenario. (a and c) The GPR as a function of gating centers for a 40%–60% and 30%–70% ground truth. (b and d) The GPR as a function of width for a 40%–60% and 30%–70% ground truth. The different color lines represent the various measurement configurations as stated in the legend. “T4cm” refers to a 4‐cm tumor size, and “A3cm” refers to a 3‐cm motion amplitude. The solid lines show the results from the unperturbed motion curves, while the dotted, dashed‐dotted, and dashed lines show the results from the 1 mm, ‐1 mm and random shifts, respectively. The impact of the random shift is very small, and the dashed and solid lines overlap completely. The green dotted line is the actual ground truth

The results of the optimizer sensitivity to small perturbations are shown in Figure 8. Due to the difficulty of visualizing the GPR as a function of the entire two‐dimensional parameters, only a cross sectional view of the GPRs at constant gating width and centers is shown. The different color lines represent the different measurement configurations with different tumor sizes and motion amplitudes. The solid line represents the unperturbed result, while the other different line styles of the same color represent the various perturbations. The unperturbed result yields the correct gating width and center with a GPR of 1.0 at the maximum point, which shows the optimizer is working as expected. The GPR of smaller tumor size and motion amplitudes tend to be higher, which is also a sensible result. In general, the maximum point is much more distinct across the different center values compared to the different width values. The maximum gating window center calculated from $pI_{\mathrm{perturb}}$ is also relatively stable with maximal variation of 10% from the ground truth. On the contrary, the optimal gating width deviates between 10% and 40% from the ground truth across the different perturbation scenario. The only exception is the result with 4‐cm tumor size and 3‐cm motion amplitude where the optimal gating width deviates by at most 10% from the ground truth. The ±1 mm translational shift perturbation also has the greatest destabilizing effect on the values of optimal gating width and center compared to the normal random errors. In fact, the dashed lines, representing the results with random errors introduced, overlapped completely with the solid lines causing it to be nonvisible in Figure 8.

## 4 DISCUSSIONS

In this work, the tumor phantom was printed with 85% infill and a linear pattern. The characterization of the 3D printing parameters results is shown in Figure 2. Moreover, Figure 2 shows that linear pattern is the fastest, and at 85% infill, it is able to give an HU closest to zero. The effects of the mean positional error of 0.0266 cm are negligible considering the magnitude of the set‐up and alignment errors of the QA phantom.

There are also higher positional errors present at the maximum and minimum point of the breathing curve stem from the finite time delay (from the sending and executing of commands), which makes it challenging to accommodate the rapid change in velocity near the turning point. As a result, the measured breathing curve “over‐travels” beyond the actual turning point position. This time delay in executing the motion is also evident from Figure 3a, and the delay results in the period of the measured breathing motion to increase by 6.55%. However, when considering the variability in actual breathing motion (both intra and inter fractions), this delay can be said to have negligible effects. This is further supported by the results in Figure 8 where the random errors based on the positional errors of the linear stages were introduced, and the GPR curves with the random errors remain indistinguishable from the unperturbed results. Overall, the entire workflow has been characterized. This was achieved by quantifying the reproducibility of the patient breathing curve during QA and printing the tumor phantom reliably.

After characterizing the proposed QA set‐up, the next step evaluates the accuracy of our algorithm (which consists of the phantom averaging of relevant phases and applying Equation (1)) in calculating $PD_{\mathrm{measured}}$. The high GPR values at the 2 mm/2% and 1 mm/1% in Figure 4 indicate good agreement between $PD_{\mathrm{measured}}$ and $PD_{\mathrm{ph}}$. The GPRs are also consistently lower in all moving phantoms compared to the static phantom. The gamma maps of the PDs in static phantom (Figure 5) show that the phantom is aligned accurately to the isocenter meaning to say that there are minimal alignment errors while setting up the QA device.

A close inspection of the gamma maps in Figure 6 shows that the agreement was excellent within the tumor phantom, and the failing pixels in the moving phantoms are at the connecting rod and the sup‐inf edges of the phantom. The gamma values in these failing pixels lies mainly between 1 and 4 when using the 2 mm/2% criteria. The positions of the failing pixels at the extreme ends of the phantom were due to a small positional offset between the centroids of the tumor phantoms in $pI_{\mathrm{measured}}$ and $pI_{\mathrm{ph}}$. There were two possible origins of this offset. Firstly, this could have been due to a slight difference between the measured breathing curve and the actual breathing curve (which is used to calculate $pI_{\mathrm{measured}}$). Secondly, there might have been a phase shift, where the real‐time phase determination during irradiation does not coincide with the phases defined in the actual breathing curve acquired during simulation. However, the impact of these factors on the result as seen in Figure 4 was minimal as the GPR is still high. Hence, this proves that our algorithm can calculate $pI_{\mathrm{measured}}$ accurately, and any discrepancy can be attributed primarily to breathing curve variation and phase shift. Any of these factors could result in tumor phantom being irradiated at the wrong position.

The statistically significant difference between the GPR of the 40%–60% and 30%–70% gating windows at 1 mm/1% can be attributed to the frequent beam holds with 40%–60% gating windows. These beam holds allowed for the introduction of more errors affecting the accuracy of the dose delivered within the gating window. This is illustrated clearly in Figure 7c where almost all the pixels (in yellow) show a higher gamma value for 40%–60% gating windows compared to 30%–70% gating windows. Significant GPR difference is not detected at the 2 mm/2% level as this criterion is less sensitive and cannot detect the small errors introduced by the increased beam holds.

Other than assessing gating accuracy with GPR and the gamma heatmap, we have developed another approach that is based on optimizing the GPR between $pI_{\mathrm{measured}}$ and $pI_{\mathrm{ph}}$ to deduce the gating window. This approach directly gives an indication of the phase gating accuracy. The algorithm is tested on a realistic patient's breathing curve, and the results in Tables 1 and 2 show that the gating center discrepancy is between 0% and 10% regardless of the size of the tumor, gating windows, and plans. A 10% discrepancy amounts to about 1‐mm positional difference in this example hence showing the tumor was still irradiated at the planned position. Unfortunately, the size of the estimated gating windows does not coincide with the size of the selected window, which can be explained by the perturbation sensitivity analysis results in Figure 8. Specifically, Figure 8b,d shows that the GPRs across different gating widths are quite similar, and the optimal gating width can easily deviate from the ground truth upon introduction of perturbations. In fact, for small tumor size and small breathing motion, the GPRs across different gating widths are indistinguishable especially when factoring in the perturbations. Thus, the failure to arrive at an accurate gating width in Tables 1 and 2 stems more from a theoretical limitation of using transit portal dosimetry framework with respiratory gating. The center of the gating window, on the contrary, is relatively stable upon perturbation with a largest 10% phase deviation from ground truth as shown in Figure 8a,c. This result coincides with the results in Tables 1 and 2 where up to 10% gating center discrepancies are observed. Overall, we have shown that our proposed method is suitable for estimating the center reliably (within the 10% uncertainty bounds) but not the width of the gating window.

Despite the convenience of using PD for phase gating QA, there are shortcomings in this approach as well. Firstly, unlike commercial phantoms, a PD detector is static and does not move with the tumor phantom. This means PD is not able to measure dose within the tumor and quantify the interplay effect.^35^, ^36^, ^37^ Secondly, the algorithm for calculating $pI_{\mathrm{ph}}$ relies on an averaging effect, which works best when the irradiation time is significantly larger than the period of the breathing curve.

This work uses a simple spherical tumor phantom as a proof‐of‐concept, and there are future developments to develop a more realistic tumor phantom using 3D‐printing.^38^, ^39^ Admittedly, this will add complexity to the $PD_{\mathrm{ph}}$ calculation especially for VMAT where the calculation must be done at the segmental level as the phantom will look differently from each beam‐eye view at a different gantry angle. Also, current work only uses a 1D motion for the tumor phantom, but this will be extended to 3D^40^, ^41^ motion in the future when extraction of the 3D motion of tumor from 4DCT would be possible. This QA approach can be easily extended for amplitude gating^42^, ^43^ as well. Additionally, there are plans to use this tool for comparing phase and amplitude gating on different patient‐specific factors such as breathing curves and tumor sizes.

## 5 CONCLUSION

A QA tool comprising a 3D printed spherical tumor phantom, programmable stages, and an EPID detector was assembled for the purpose of testing the phase gating accuracy with RPM systems. An algorithm was also developed to estimate $pI_{\mathrm{ph}}$ in moving phantom, which has shown to agree with high GPR with experimental data under different gating windows, phantom sizes, and plans. Finally, using an actual patient's breathing curve and plan this work proved that the center of the gating window can be determined accurately with the proposed QA device and algorithm.

## CONFLICT OF INTEREST

The authors have no conflict of interest to disclose.

## AUTHOR CONTRIBUTIONS

Hong Qi Tan involved in conceptualization, methodology, investigation, data analysis, and writing original manuscript. James Cheow Lei Lee and Sung Yong Park involved in conceptualization and reviewing the manuscript. Calvin Wei Yan Koh, Kah Seng Lew, Clifford Ghee Ann Chua, and Khong Wei Ang involved in methodology, investigation, and reviewing the manuscript.
